# Semaphorin 3A Is Effective in Reducing Both Inflammation and Angiogenesis in a Mouse Model of Bronchial Asthma

**DOI:** 10.3389/fimmu.2019.00550

**Published:** 2019-03-22

**Authors:** Sabag D. Adi, Nasren Eiza, Jacob Bejar, Hila Shefer, Shira Toledano, Ofra Kessler, Gera Neufeld, Elias Toubi, Zahava Vadasz

**Affiliations:** ^1^Proteomic Unit, The Division of Clinical Immunology and Allergy, Bnai-Zion Medical Center, Haifa, Israel; ^2^The Department of Pathology, Faculty of Medicine, Bnai-Zion Medical Center, Haifa, Israel; ^3^The Ruth and Bruce Rappaport Faculty of Medicine, Technion Israel Institute of Technology, Haifa, Israel

**Keywords:** semaphorin3A, asthma, inflammation, angiogenesis, BAL (bronco-alveolar lavage)

## Abstract

Semaphorin 3A (sema3A) belongs to the sub-family of the immune semaphorins that function as regulators of immune-mediated inflammation. Sema3A is a membrane associated molecule on T regulatory cells and on B regulatory cells. Being transiently ligated to the cell surface of these cells it is suggested to be a useful marker for evaluating their functional status. In earlier studies, we found that reduced sema3A concentration in the serum of asthma patients as well as reduced expression by Treg cells correlates with asthma disease severity. Stimulation of Treg cells with recombinant sema3A induced a significant increase in FoxP3 and IL-10 expression. To find out if sema3A can be of benefit to asthma patients, we evaluated the effect of sema3A injection in a mouse model of asthma. BALB\c-mice were sensitized using ovalbumin (OVA) + adjuvant for 15 days followed by OVA aerosol inhalation over five consecutive days. Four hours following air ways sensitization on each of the above days- 15 of these mice were injected intraperitoneally with 50 μg per mouse of recombinant human sema3A-FR and the remaining 15 mice were injected with a similarly purified vehicle. Five days later the mice were sacrificed, broncheo-alveolar lavage (BAL) was collected and formalin-fixed lung biopsies taken and analyzed. In sema3A treated mice, only 20% of the bronchioles and arterioles were infiltrated by inflammatory cells as compared to 90% in the control group (*p* = 0.0079). In addition, eosinophil infiltration was also significantly increased in the control group as compared with the sema3A treated mice. In sema3A treated mice we noticed only a small number of mononuclear and neutrophil cells in the BAL while in the control mice, the BAL was enriched with mononuclear and neutrophil cells. Finally, in the control mice, angiogenesis was significantly increased in comparison with sema3A treated mice as evidenced by the reduced concentration of microvessels in the lungs of sema3A treated mice. To conclude, we find that in this asthma model, sema3A functions as a potent suppressor of asthma related inflammation that has the potential to be further developed as a new therapeutic for the treatment of asthma.

## Introduction

Semaphorins were initially identified as axon guidance factors but have subsequently been characterized in addition as modulators of angiogenesis, and as modulators of immune responses. The involvement of some semaphorins such as sema3A and sema4D in both innate and adaptive immune responses resulted in their characterization as a semaphorin subgroup of “immune semaphorins” (1). Semaphorin 3A (sema3A), is a member of the secreted class-3 semaphorins (2). Following secretion sema3A binds to the neuropilin-1 receptor which in association with receptors of the plexin family form functional sema3A receptors in responsive cell types (2). Sema3A had been characterized as a regulator of immune mediated inflammation. Incubation of sema3A with stimulated T effector cells inhibits their proliferation and their ability to secrete pro-inflammatory cytokines (3). The transient ligation of sema3A on Tregs from patients suffering from rheumatoid arthritis (RA) is decreased in association with increased disease activity (4). Taken together, these findings establish sema3A as an indicator for Treg cells activation and as a target for the development of therapeutics targeting inflammatory diseases. When recombinant sema3A was injected into collagen-induced arthritic (CIA) mice, Treg cell function was restored and RA disease activity in these mice was inhibited (4). The concentration of sema3A was found to be reduced in serum of systemic lupus erythematosus (SLE) patients and in correlation with SLE disease activity (5). Furthermore, injection of sema3A into NZB/W mice (an animal model of SLE) reduced and delayed proteinuria, renal damage and prolonged the survival of these mice (6).

Airway inflammation in asthma patients is a complex process, mainly characterized by T helper-2 cells (Th2) hyper-activation. Consequently they display enhanced responses to environmental allergens, and over-produce pro-inflammatory cytokines. This is followed by the activation of allergen-specific B cells and the production of high amounts of specific IgEs, leading to mast cell degranulation (7). The concentration of Treg cells, as well as FoxP3 expression and IL-10 production are deficient in asthma thereby contributing to airway inflammation and disease activity. Upon treatment with corticosteroids and following Allergen Immunotherapy (AIT) the concentrations of the Treg cells and the expression of FoxP3 and IL-10 can be restored and clinical improvement of asthma achieved (8, 9). Subsequently, the concentration of T regulatory cells (CD4/CD25/highFoxP3+) were found to be reduced in induced sputum of atopic asthmatics found to be negatively correlated with airway hyper-responsiveness (10). In patients with active bronchial asthma, decreased amounts of Treg cells and altered expression of FoxP3 were found to be associated with increased level of Th17 cells. In this case, dexamethasone therapy was shown to correct this disturbed balance between Treg and Th17 cells (11). Chronic airway structural changes such as smooth muscle cells hypertrophy and angiogenesis are consequences of long-lasting inflammation in bronchial asthma and are considered to be part of the remodeling process (12). In contrast with the beneficial effect of inhaled corticosteroids in reducing lung T cell and eosinophil infiltration, it is still unclear if inhaled corticosteroids inhibit angiogenesis in bronchial asthma. Sema3A is a membrane associated molecule on Tregs and the newly defined Breg cells. Sema3A plays a regulatory role in experimental mouse models as well as in human models of allergic rhinitis, atopic dermatitis and asthma (13). We have found that low serum levels of sema3A are correlated with severe asthma. Incubation of recombinant sema3A with Treg cells increased the expression of FoxP3 in normal individuals but less so in Tregs of asthmatics (14). We assume that this regulatory effect of Sema3A on Treg cells is via its ligation to neuropilin-1, the known functional receptor of Sema3A on Treg cells. With this in mind we have evaluated in the present study the therapeutic immune-modulatory effects of sema3A following its injection into mice in which we have induced asthma using ovalbumin (OVA) adjuvant. We find that in this asthma model, sema3A functions as a potent suppressor of asthma related inflammation that in addition inhibits asthma associated angiogenesis.

## Materials and Methods

### Production and Purification of Recombinant Point Mutated Human Furin Cleavage Resistant sema3A

The construction of a lentiviral expression vector directing the expression of point mutated furin like pro-protein convertases resistant sema3A containing a c-terminal 6xHis epitope tag (FR-sema3A) was previously described (15). Lentiviruses directing expression of FR-sema3A or control empty lentiviruses were used to infect HEK-293 cells. Conditioned medium from FR-sema3A producing and from control cells, was purified on nickel columns according to the manufacturer instructions. The purified FR-sema3A (FR-sema3A) and the corresponding fractions eluted from nickel columns loaded with control conditioned medium (vehicle) were then used in subsequent experiments.

### Asthma Mouse Model

Thirty female Balb/c mice 6- to 7-old weeks were included in this study. OVA sensitization and airway challenge were performed as follows: the mice were sensitized intraperitoneally with 50 μg ovalbumin (OVA; grade V; Sigma-Aldrich) emulsified in 2 mg Alum-Hydroxide (Sigma-Aldrich) in 200 μl 0.9% sodium chloride (saline; Hospira) on Days 0, 7, and 14. On Days 22–25, the mice were placed in a box and were exposed each day for 20 min to an aerosol consisting of 1% (m\v) OVA dissolved in PBS, Ph-7.4 (16). Four hours following air ways sensitization on each of the above days- 15 of these mice were injected intraperitoneally with 50 μg per mouse of recombinant human sema3A-FR and the remaining 15 mice were injected with a similarly purified vehicle as described above. The mice were sacrificed 5 days after sema3A injection on day 30. Broncho-alveolar lavage (BAL) was collected and lung tissue taken for evaluation of treatment efficacy.

### Inflammatory Cells in BAL

The BAL fluid was centrifuged at 2,000 rpm for 10 min. After discarding the supernatant the sediment was fixed on lysine-coated slides (Leica Biosystems, Germany), dried and stained with hematoxilin-eosin. The total number of inflammatory cells was counted double blindly by two expert pathologists. They then scored these numbers to “grade”; above 100 cells\slide- graded as “3,” 50–100 cells\slide- graded as “2,” 10–50 cells\slide- graded as “1” and <10 cells\slide-graded as “0.” The results are the average of these results.

### Lung Biopsies

Lungs were formalin-fixed and paraffin embedded (FFPE). Five microns slides were cut and subjected to hematoxylin-eosin staining. The evaluation of the extent of the inflammatory process around blood vessels and bronchioles and the evaluation of the number of eosinophils per high power microscopic field (HPF) was performed by two expert pathologists. The results are expressed as the average of these results.

### The Anti-angiogenic Effects of sema3A

Sections of the FFPE samples were mounted onto electrostatically charged microscope slides, dried at 60°C for 1 h, dewaxed and rehydrated as follows: Twice in 100% Xylene for 5 min, twice in 100% Ethanol for 5 min, once in Methanol+H_2_O_2_ for 20 min, once in 70% Ethanol for 5 min, once in 50% Ethanol for 5 min, once in 25% Ethanol for 5 min, and twice in distilled water (DW). The slides were transferred to a working surface and incubated in warm Trypsin-PBS 1:1 for 2 min then washed in PBSx1. The slides were then transferred into retrieval solution-10 mM Tris buffer PH = 8 and put in the microwave for 18 min, then cooled on the bench and washed with DW. The slides were blocked with 10% normal goat serum (NGS) for 1 h at room temperature, and incubated in primary antibody (rat anti-mouse CD31,Dianova, Hamburg, Germany, 1:100 diluted in 5% NGS) overnight at 4°C. The next day the slides were washed 3 × 5 min in PBS, incubated in secondary antibody conjugated to biotin(diluted in 5% NGS) for 1 h at room temperature, 3 × 5 min washed in PBS, then incubated in HRP Streptavidin antibody (diluted in 5% NGS) for 1 h in RT and washed 3 × 5 min in PBS. The slides were then stained with 3-amino-9-ethylcarbazole (AEC) staining for 15 min, washed in DW and stained with Hematoxylin for 30 s. The evaluation of angiogenic blood vessels in the tissue was performed by two expert pathologists and the results are the mean of their evaluation.

### Statistical Analysis

A comparison between two groups was performed using the Mann–Whitney non-parametric test. A two-tailed *P*-value of 0.05 or less was considered to be statistically significant.

## Results

### The Effect of sema3A Treatment on the Concentration of Inflammatory Cells in BAL

**Figure 1:**
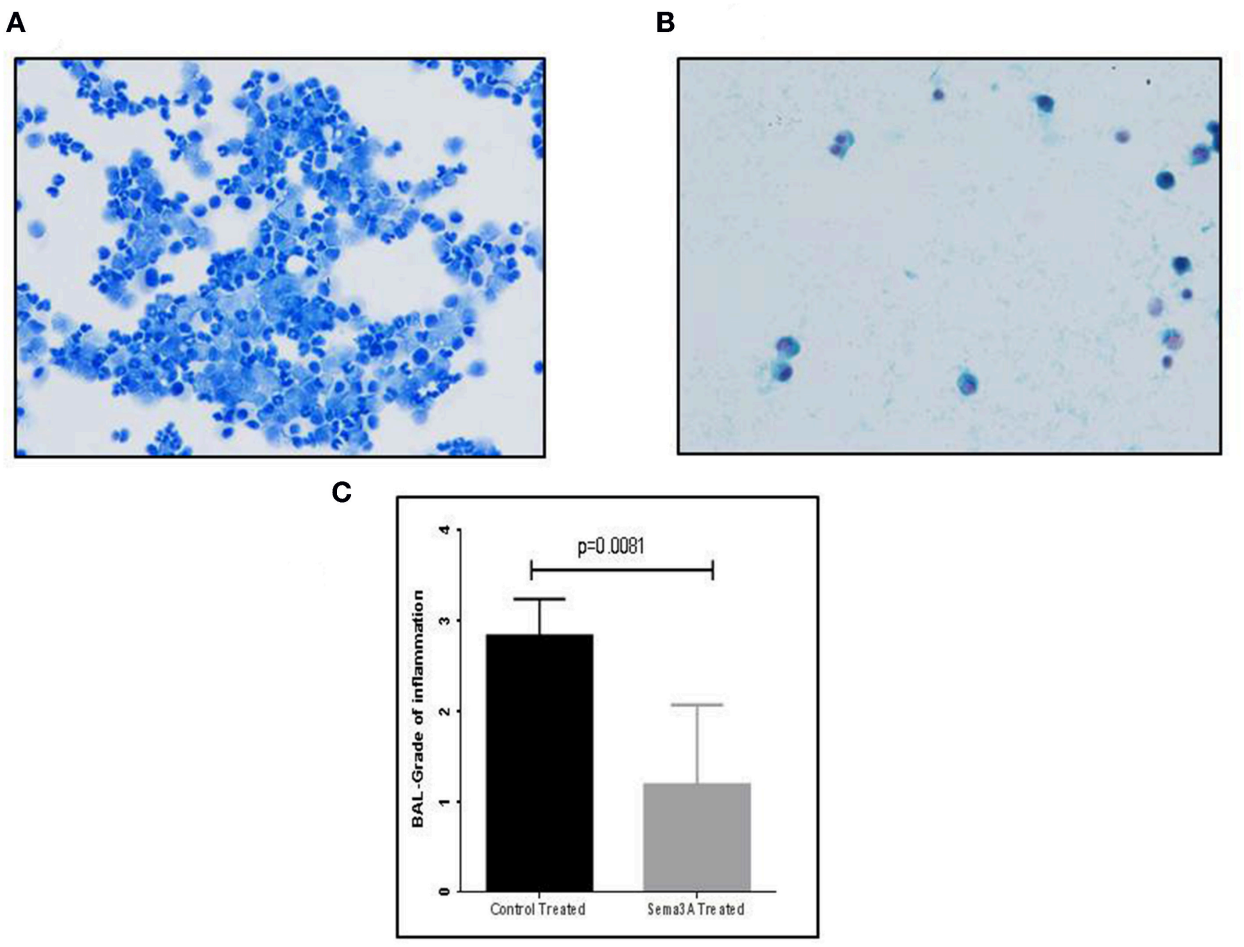
The effect of sema3A treatment on the concentration of inflammatory cells in the BAL. **(A)** A representative picture of inflammatory cells in the BAL fluid of vehicle treated mice. **(B)** A representative picture of inflammatory cells in the BAL fluid of sema3A treated mice. **(C)** Comparison of the average grade of inflammatory cells in the BAL of vehicle vs. sema3A treated mice as determined by the counting of 3–5 microscope fields derived from 15 mice in each group followed by grading as described in materials and methods. Error bars represent the standard error of the mean. Statistical significance was determined using the Mann–Whitney non-parametric test.

Mice were sensitized by OVA adjuvant injection followed by OVA inhalation to generate asthmatic mice. Mice were then divided into two groups and treated by injection of vehicle or sema3A as described in materials and methods. After 5 days the concentration of inflammatory cells in BAL fluid derived from vehicle treated mice or sema3A treated mice was compared by analysis of microscopic fields followed by grading as described in materials and methods. The BAL of sema3A treated mice contained a low concentration of inflammatory cells while the BAL of vehicle treated mice contained a significantly higher concentration (*P* = 0.0081) of inflammatory cells (see Figure 1).

### The Effect of sema3A Treatment on Lung Inflammation and Eosinophils Induced by OVA Sensitization in Mice

**Figure 2:**
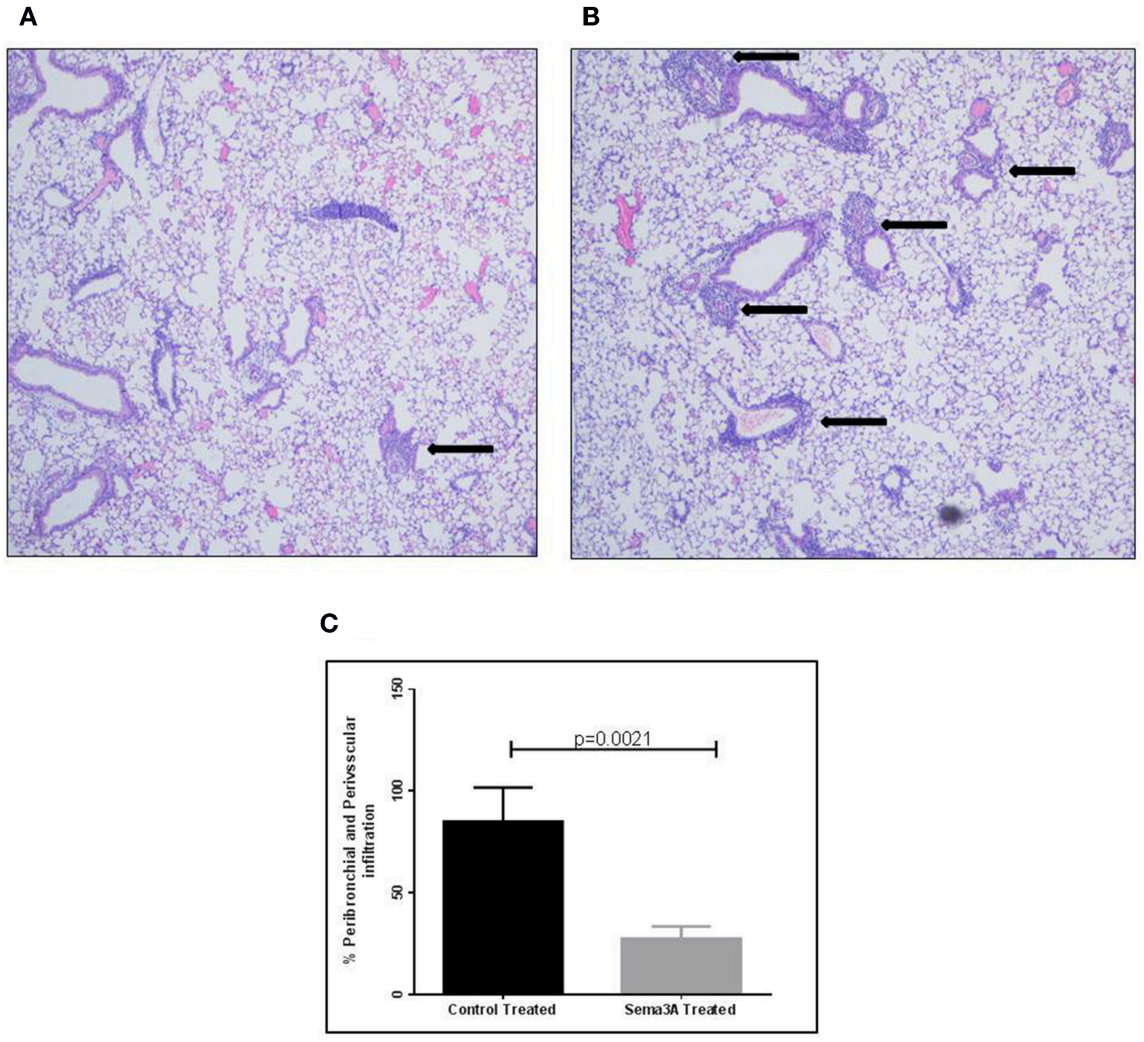
Sema3A inhibits the infiltration of inflammatory cells into the lungs of OVA sensitized asthmatic mice. **(A)** Infiltration of inflammatory cells around bronchiole, in sema3A treated mice. Black arrow denotes inflammation (Magnification X20). **(B)** Infiltration of inflammatory cells around bronchioles and blood vessel, in vehicle treated mice. Black arrows denote massive inflammation (Magnification X20). **(C)** Percentage of bronchioles and blood vessels that are surrounded by inflammatory cells in HPF. The results are the mean value of 3–5 HPF, derived from 15 mice in each group.

**Figure 3:**
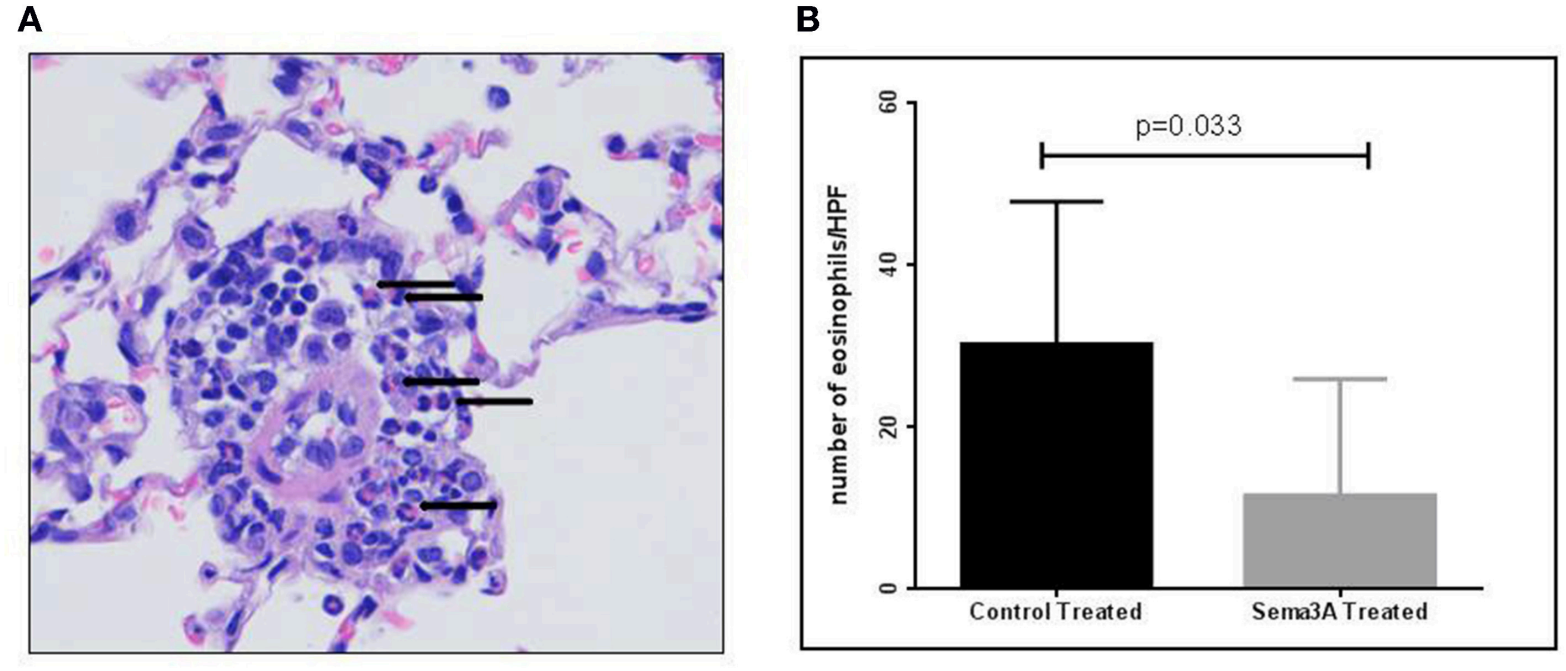
Sema3A inhibits eosinophil infiltration into the lungs of OVA sensitized asthmatic mice. **(A)** Representative figure of eosinophil infiltrate in lungs of OVA sensitized asthmatic mice. Black arrows denote eosinophils (Magnification X40). **(B)** Number of eosinophils per HPF in the inflammatory cell infiltrates in vehicle vs.sema3A treated mice. The results are the average of 3–5 HPF, derived from 15 mice in each group.

Inflammation in tissues surrounding the bronchioles and blood vessels in lungs of mice sensitized by OVA and then treated with sema3A or vehicle was determined in histological sections obtained from the lungs of the mice. In sema3A treated mice only few bronchioles (25 ± 10%) were surrounded by invading inflammatory cell (Figures 2A,C) while in lungs derived from control mice most of the bronchioles (85 ± 10%) attracted inflammatory cells (*P* = 0.0021) (Figures 2B,C). The infiltration of eosinophils (Figure 3A, black arrows) was also compared between the two groups and was also more pronounced in the control group with 25 ± 8 eosinophils per HPF as compared with only 5 ± 2 eosinophils per HPF in the sema3A treated mice (*P* = 0.033) (Figure 3B).

### Sema3A Inhibits Angiogenesis Induced by OVA Sensitization in the Asthmatic Mouse Model

**Figure 4:**
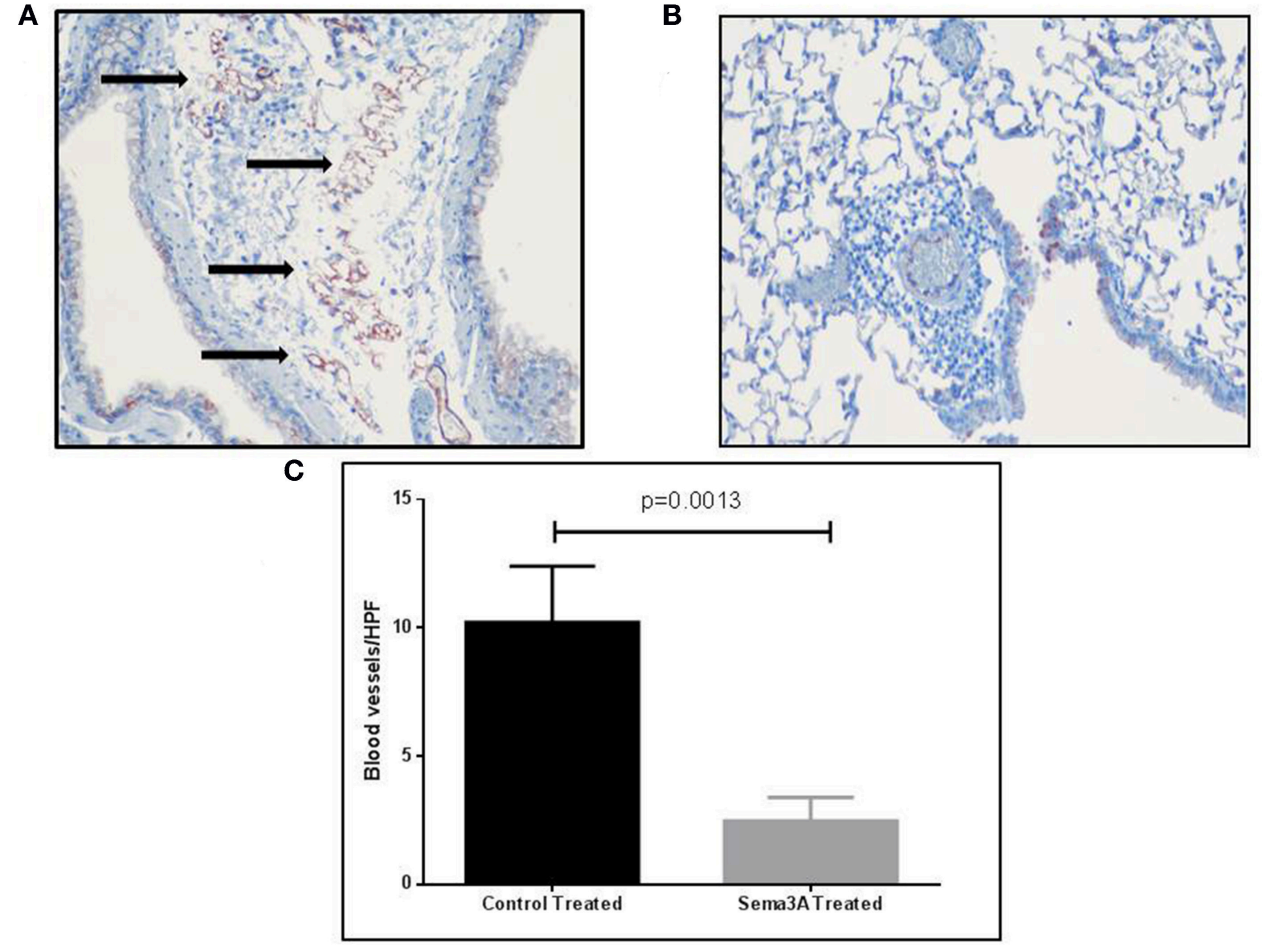
The effect of sema3A on angiogenesis induced by OVA sensitization in lungs of mice. **(A)** Blood vessels in the lung tissue in vehicle treated mice were stained with an antibody directed against CD31 as described in materials and methods. Shown is a representative HPF. Black arrows denote microvessels. **(B)** Shown is a representative HPF. Blood vessels in the lung tissue in sema3A treated mice were stained similarly. Almost no blood vessels can be seen. **(C)** Quantification of the blood vessels concentration across all examined histological slides in vehicle treated and in sema3A treated mice (15 mice in each group, *P* = 0.0013).

Sema3A is a potent anti-angiogenic factor (17). We have therefore determined if sema3A can inhibit angiogenesis induced by OVA sensitization in the lungs of mice (18). Indeed the lungs of mice treated with sema3A contained a significantly reduced concentration of micro-vessels as compared to lungs of control mice (Figures 4A,B). In the vehicle-treated group the concentration of micro-capillaries was 10.22 ± 2.178\HPF as compared with a concentration of 2.46 ± 0.932 capillaries\HPF in lung biopsies derived from sema3A treated mice (*p* = 0.0017) (Figure 4C).

## Discussion

Airway inflammation in bronchial asthma is classically characterized by multiple inflammatory pathways involving both innate and adaptive immune responses. The hallmark of this inflammation is considered to be T-cell-driven, involving all T cell phenotypes. Increased IL-17 production was found to be responsible for the neutrophil influx into airways as well as for the depletion of Tregs in peripheral blood and in the inflamed airways of patients with bronchial asthma. IL-22 was also reported to be involved in airway hyper-reactivity and in the inflammation of asthmatic OVA-sensitized mice. Lungs of such mice were infiltrated with CD3+CD4+IL-22+T cells that co-expressed IL-17 and TNF-α in association with neutrophil airway infiltration (19–21). The involvement of Th2-type cytokines such as IL-4 and IL-5 was also reported to be important in the pathogenesis of asthma, and was found to shift the differentiation of naïve CD4+ T cells into Th2 cytokine-producing eosinophils and to promote eosinophil infiltration into the inflamed bronchial tree (22). Current therapeutic approaches in bronchial asthma are directed against all of the above-mentioned inflammatory pathways. They classically include corticosteroids (both inhaled and systemic), that are mainly beneficial in altering neutrophil and eosinophil infiltration in the bronchi. When steroids are insufficient, newly introduced anti-IgE or anti-IL-5 drugs are of additive value and reported to be effective in sparing steroids (23, 24). A new approach focused on the modulation of relevant regulatory pathways rather than on the use of immunosuppressive agents (such as steroids and cytotoxic drugs), for the treatment of immune-mediated inflammatory diseases is gaining popularity. In this context, sema3A was recently reported to be a good candidate (6). In previous studies we demonstrated the beneficial effect of sema3A as a down-regulator of the increased expression of TLR-9 in activated B cells from both normal individuals and from patients suffering from SLE (25). Subsequently we also found that sema3A increases the expression of CD72 (a regulatory molecule) on B cells, in addition to enhancement of Treg cell functions (26). These findings are in accordance with *in-vivo* studies in which the injection of recombinant sema3A effectively improved allergic rhinitis and atopic dermatitis in relevant mice models. In these models sema3A was shown to improve both clinical symptoms and tissue inflammation (27, 28). The present study is the first to show that sema3A also reduces efficiently the infiltration of both neutrophils and eosinophils in lung tissue inflammation and in BAL derived from OVA-sensitized mice. We assume that this effect is achieved by increasing local Treg functions, which subsequently reduces adaptive immune-mediated responses and pro-inflammatory cytokines. It is also possible that sema3A inhibits IL-17 production, indirectly leading to the reduced influx of neutrophils, although experimental proof for that is still required (29). So far there is no evidence for the presence of sema3A receptors on neutrophils and eosinophils. Long-lasting airway inflammation may lead to structural changes termed remodeling. These changes consist of sub-epithelial layer thickening, airway smooth muscle hyperplasia and increased angiogenesis induced by the expression of angiogenic factors such as VEGF and angiopoietin (30). Ongoing angiogenesis in the alveoli of asthmatic patients is usually followed by tissue edema and increased vascular permeability which is also triggered by VEGF. Inhaled corticosteroids and anti-leukotriens are of limited influence on angiogenesis and remodeling in most cases. The timing of anti-angiogenic therapy is crucial in attenuating this process and preventing irreversible tissue remodeling. The effect of biological therapies such as the anti-IgE omalizumab and the anti-IL-5 mepolizumab on remodeling is still ill defined and remains to be assessed (31, 32). Thus, new anti-angiogenic compounds such as sema3A are needed in order to control remodeling in asthma. Of the reported mechanisms by which sema3A inhibits angiogenesis, the most important is its high efficacy in inhibiting VEGF activity as a result of the activation of inhibitory intracellular pathways that inhibit VEGF signal transduction (33). Sema3A may also inhibit airway smooth muscle cell proliferation (34) and may in addition reduce angiogenesis by reducing expression of nitric oxide (NO). Diminished NO production was found to be in association with increased smooth muscle cell hyperplasia and with vascular remodeling of blood vessels. Reduced NO production was associated with reduced NO production in patients suffering from pulmonary vascular diseases thus supporting a possible role for NO deficiency in vascular remodeling (35, 36). This observation suggests that improved eNOS-NO pathway signaling may represent a beneficial outcome when considering possible sema3A therapy. The full understanding of all mechanisms by which sema3A decreases eosinophil infiltration in lung tissues and by which it inhibits angiogenesis is still ill defined. To summarize, our experiments indicate that sema3A should be considered as a possible novel therapeutic agent for the treatment of bronchial asthma. Future studies should focus on the strengthening of these results by demonstration of the benefit of sema3A for the improvement of lung functions in asthmatics.

## Ethics Statement

This study was carried out in accordance with the recommendations of Ministry of Health-Israel-Ethical license. The protocol was approved by the Ministry of Health-Israel-Ethical committee.

## Author Contributions

ZV and ET conducted this research, participated in the experiments, and were responsible for this manuscript writing and editing. SA, NE, OK, and GN were responsible for analysis of results and participated in the experiments. JB and HS participated in the histological analysis of results. ST contributed to the manufacturing and purification of recombinant human sema3A.

### Conflict of Interest Statement

The authors declare that the research was conducted in the absence of any commercial or financial relationships that could be construed as a potential conflict of interest. The handling editor declared a shared affiliation, though no other collaboration, with several of the authors GN, ST, and OK.
